# dsRNA as broad-spectrum antiviral therapeutics

**DOI:** 10.1016/j.omtn.2022.09.023

**Published:** 2022-10-19

**Authors:** Congfei Xu

**Affiliations:** 1 School of Biomedical Sciences and Engineering, South China University of Technology, Guangzhou International Campus, Guangzhou 511442, P.R. China

The human immune system can detect the presence of foreign double-stranded RNA (dsRNAs) and activates the innate immune response against viral invaders by boosting the secretion of cytokines called interferons (IFN). In this issue, Si et al. have found a new class of immunostimulatory dsRNAs that potently promote the production of two kinds of IFN (IFN-I and IFN-III) while reducing the inflammation often observed with known types of immunostimulants.^1^ When the new dsRNA molecules were tested in traditional cell culture as well as a complexed Human Organ Chip model of the lung that faithfully mimics its *in vivo* counterpart, they inhibited infection by many viruses with pandemic potential, such as SARS-CoV, SARS-CoV-2, MERS-CoV, and various influenza A strains. In a mouse model of COVID-19, the dsRNA decreased the virus by more than 1,000-fold. This technology has the potential to become a broadly applied approach for combating future pandemics.

The discovery was made inadvertently when short interfering RNA (siRNA) sequences were developed to "knock down" host genes and determine how this affected influenza viral infection. The researchers discovered that a mixture of three siRNAs prevented influenza A infection of lung cells by more than 90% and lowered a gene named *DGCR5* by more than 80%. However, subsequent analysis revealed that this effect was a side effect of the siRNA and not a direct effect of *DGCR5*. This specific siRNA sequence, which is a dsRNA of 25–27 nucleotides, induced the overexpression of numerous genes involved in the IFN signaling pathway. This was particularly noteworthy due to the fact that the siRNA sequences they were employing were purposefully devoid of recognized immune-stimulating structural characteristics. Additional study indicated that this particular siRNA exclusively triggered the RIG-I pathway, but not the other two known downstream IFN signaling pathways (TLR3 or MDA5). To better understand the mechanism of action of this type of previously unidentified immunostimulatory RNA, more than 200 dsRNA sequences were then generated and tested to tease out what was driving the IFN stimulation. They identified a particular nucleotide sequence motif, which occurred on the ends of two similar dsRNAs with high IFN activity: a cytosine (C) on one strand and three guanines (GGG) on the other strand. When this motif’s sequence or position within the molecule was modified, the dsRNAs lost their immunostimulatory effects, confirming that it was the crucial element driving IFN activation. Because the C and the GGG elements in the motif are different lengths, the end of each dsRNA molecule has an “overhang” of two guanines. When many copies of the dsRNA are present, four of these guanines can bind to each other via an unusual phenomenon called G-G Hoogsteen base pairing. The resulting dimers of dsRNA can then bind to RIG-I more effectively, causing its activation.

To determine the efficacy of their newly found dsRNAs in living cells, the researchers pitted one of them against poly (I:C), a widely used immunostimulant that can activate many cellular sensors, including TLR3, RIG-I, and MDA5. When administered to human epithelial cells, dsRNA produced a more restricted, less inflammatory antiviral response, whereas poly (I:C) caused far larger changes in gene expression and affected other biological processes needed for normal cell function.

Subsequently, the team then tested the dsRNA in a Human Lung Airway Chip and Alveolus Chip that replicate human lung function and responses to viral infections *in vitro*.^2^, ^3^, ^4^ They transfected the dsRNA into cells in healthy chips and saw a 12- to 40-fold increase in IFN-beta expression. When influenza A was then injected into the organ chips, they discovered that the dsRNA inhibited influenza A infections by 80%–90%.

The COVID-19 pandemic made it evident that we need broad-spectrum therapies that can attenuate infection by a wide range of viruses, rather than designing a tailored therapy for each illness as it emerges. So, the researchers tested the efficacy of the dsRNA against related coronaviruses SARS-CoV, MERS-CoV, and HCoV-NL63. In a mammalian cell line, their dsRNA inhibited MERS-CoV and HCoV-NL63 by more than 90%, and SARS-CoV by more than 1,000-fold. Furthermore, it prevented SARS-CoV-2 infection in human epithelial cell lines by 99.99%. Finally, the dsRNA was evaluated in a mouse model of COVID-19. When mice were infected with SARS-CoV-2, the dsRNA lowered the viral burden by more than a factor of 1,000 when delivered intravenously.

In addition to treating viral infections, this class of dsRNAs has the potential to treat bacterial, fungal, and parasite infections, as well as diseases such as cancer that could benefit from an increase in IFN production. Additionally, they could be employed as an adjuvant to boost the efficacy of other vaccinations. However, future research is required to identify the best time of treatment, as activating IFN too late in an infection may aggravate inflammation. Better routes of delivering the dsRNA to reduce systemic immune activation., for example, directly to the upper airway, is also needed to translate into human treatment.
